# Comment on: Machine learning-based prediction of short- and long-term mortality for shared decision-making in older hip fracture patients

**DOI:** 10.2340/17453674.2026.45362

**Published:** 2026-04-08

**Authors:** Eda Yıldırım

**Affiliations:** Department of Orthopedics and Traumatology, Başkale State Hospital, Van, Turkey

*Sir,* — I recently read with great interest the article by Dijkstra et al. entitled “Machine learning-based prediction of short- and long-term mortality for shared decision-making in older hip fracture patients: the Dutch Hip Fracture Audit algorithms in 74,396 cases” [1]. The authors are to be commended for using a large national registry to develop interpretable prediction models and for explicitly positioning these tools as supportive instruments rather than determinants of clinical decision-making.

I fully agree with the authors that mortality prediction models should not be used in isolation and cannot replace clinical judgment. I would like to further reflect on how such prognostic information is interpreted and applied within the shared decision-making (SDM) process in daily clinical practice.

SDM is a collaborative process in which clinicians, patients, and their relatives integrate the best available evidence with individual values, preferences, and goals of care [2]. In older patients with hip fractures, treatment decisions are rarely guided by survival probabilities alone. Expectations regarding postoperative mobility, independence, and the ability to return to a meaningful level of function often play a central role in determining whether an intervention is perceived as acceptable by patients and their families. From this perspective, mortality prediction represents only one dimension of a much broader decisional landscape.

Importantly, survival does not necessarily equate to a favorable outcome after hip fracture. Numerous studies have shown that functional recovery, walking ability, and independence in activities of daily living are among the most relevant outcomes for older patients [3,4]. While the authors appropriately focus on mortality as a robust and measurable endpoint, the absence of functional outcomes may limit the ability of prediction models to fully support patient-centered SDM discussions.

In addition, the communication of algorithm-derived mortality risk deserves careful attention. Even when clinicians acknowledge that such estimates are probabilistic rather than deterministic, high predicted mortality may unintentionally influence treatment recommendations or patient perceptions. Previous work has demonstrated that prognostic information, if not carefully contextualized, can unintentionally bias decision-making toward overly pessimistic or nihilistic approaches [5]. Within SDM, it is therefore essential that mortality risk estimates are framed as supportive information and explicitly balanced with clinical judgment and patient-defined priorities.

*In conclusion,* the work by Dijkstra et al. represents an important contribution to data-informed hip fracture care [1]. Its clinical impact may currently be limited and should be further strengthened by continued emphasis on how mortality prediction models are embedded within SDM conversations and by future integration of functional and patient-reported outcomes. Such an approach may better align predictive analytics with what matters most to older patients facing complex treatment decisions after hip fracture.

**Eda Yıldırım**

*Department of Orthopedics and Traumatology, Başkale State Hospital, Van, Turkey*

*Correspondence: yldrmeddaa@gmail.com*
